# The Impact of Vision Impairment (IVI) Questionnaire; Validation of the Thai-Version and the Implementation on Vision-Related Quality of Life in Thai Rural Community

**DOI:** 10.1371/journal.pone.0155509

**Published:** 2016-05-18

**Authors:** Mansing Ratanasukon, Jongjit Tongsomboon, Patama Bhurayanontachai, Pichai Jirarattanasopa

**Affiliations:** 1 Department of Ophthalmology, Faculty of Medicine, Prince of Songkla University, Hat Yai, Songkhla, Thailand; 2 Department of Nursing, Faculty of Medicine, Prince of Songkla University, Hat Yai, Songkhla, Thailand; University of Florence, ITALY

## Abstract

The objective of this study is to validate the Thai-version of the impact of vision impairment (IVI) questionnaire and to evaluate its impact on vision-related quality of life (VRQoL) in southern Thailand. The IVI questionnaire was translated into Thai according to WHO translation guidelines. In addition to the routine ophthalmological examinations, a Thai version of the IVI questionnaire was administered to all participants. A total of 120 patients with visual impairment who presented at Songklanagarind hospital, Songkhla province, were enrolled in the study; 30 had age-related macular degeneration (AMD), 30 had cataract, 30 had diabetic retinopathy, 30 had glaucoma, and 30 non-visually impaired individuals comprised the control group. Statistical analysis demonstrated the Thai-version IVI questionnaire is valid and reliable to evaluate the VRQoL of the Thai patients through three subscales: (i) mobility and independence, (ii) reading and accessing information, and (iii) emotional well-being. The results demonstrated high consistency in all subscales with Cronbach’s alpha ranging from 0.787 to 0.849. Rasch analysis revealed the validity of the Thai-version IVI to assess VRQoL through all three subscales. Test-retest reliability was also high (intraclass correlation coefficient = 0.96). The composite score of the IVI was significantly higher in participants with visual impairment compared with healthy participants. Moreover, the subscale scores of reading and accessing information, and emotional well-being were highest in participants with AMD. While the subscale scores of mobility and independence were highest among those with either cataracts or diabetic retinopathy. The symptoms of the common vision impairment diseases are associated with an adverse impact on VRQoL in a clinic-based population as demonstrated in this study.

## Introduction

The patient-reported outcomes is commonly used in ophthalmic research and clinical practice to assess the impact of impaired vision from the patient’s perspective on patients’ quality of life (QoL) [1]. Several studies using various psychometric instruments showed the association of vision impairment and detrimental effects on vision-related quality of life (VRQoL) including daily functioning, falls, mobility, depression, life stress and satisfaction [2–4]. However, due to the unavailability of a psychometrically valid Thai language VRQoL measure, few studies have assessed the impact of vision impairment in the Thai population with vision impairment diseases.

The Impact of Vision Impairment (IVI) questionnaire is a vision specific instrument used to measure the impact of vision impairment on specific aspects of QoL and was developed using focus group discussions and input from existing instruments [3]. The IVI questionnaire has been shown to be reliable and responsive to interventions and has been rigorously validated in different ocular conditions and different levels of visual ability [5–8]. It is composed of 3 subscales: (i.) reading and accessing information (9 items), (ii.) mobility and independence (11 items), and (iii.) emotional well-being (8 items). These 3 subscales are relatable, easy to understand and easily evaluated in our Thai patients, especially those who live in the rural community.

In this study, we determined the validity, reliability, and measurement characteristics of the Thai-version of the IVI questionnaire. We also investigated the impact of the questionnaire on VRQoL in Thai patients with various vision impairment diseases.

## Materials and Methods

### Development of the Thai-version of the IVI questionnaire

The IVI questionnaire composes of 28 items grouped in three specific subscales: reading and accessing information, mobility and independence, and emotional well-being (S1 Table). The active response options of each item is expressed on a scale ranging from 0 (not at all), 1 (a little), 2 (moderately) to 3 (a lot) and items 1 to 15 have an additional response for not applicable (don't do this activity for other reasons). The Thai-version of IVI questionnaire was developed according to the WHO translation guidelines for research instruments [9]. After the professional translation from English into Thai and back translation into English, the cognitive debriefing test by ten Thai ophthalmic patients was performed to assess their understanding and interpretation of the questionnaire. The results of the pilot-testing indicated that the instrument was well accepted, and all items were easy to understand. However, the proper adaptation of the questionnaire to the Thai culture and lifestyle mandated slight modification of one question. Thus, in recreational activities “bowling, walking or golf” was changed to “walking, jogging, patong or aerobics”. The final version of the Thai-version IVI questionnaire was established after these minor revisions (S2 Table).

### Study design and subject groups

The study was approved by the research ethical committee of the Faculty of Medicine, Prince of Songkla University. All investigations were performed according to the guidelines of the Declaration of Helsinki and all participants gave informed written consent. Patients presenting for follow-up or visual consultations that met the eligibility criteria were recruited from the out-patients clinic at Songklanagarind hospital. Sample size was set at 30 subjects per group according to the calculations based upon a ratio of five cases to one item is adequate [10]. The final Thai-version IVI questionnaire was read and answered by 120 participants who had at least one of the following diseases; age-related macular degeneration (AMD) including polypoidal choroidal vasculopathy (PCV), cataracts, diabetic retinopathy, or glaucoma. The diseases were diagnosed by an ophthalmologist. In addition to these patients, 30 healthy individuals with no chronic ocular diseases, including those with refractive errors corrected with glasses or contact lens, were included in the study. Control subjects had a presenting visual acuity (VA) of better than 20/40 in the better eye. The exclusion criteria for participants are barrier to study due to illiteracy or neurological diseases affecting cognitive functions. In the first step, after explaining the aims and nature of the study, all 150 participants were requested to fill up the questionnaire by themselves. Then, to evaluate the reliability and perform test-retests, 30 patients with cataracts and 30 healthy normal were selected to complete the questionnaire again within the following 4–7 weeks.

### Statistical analysis

The Rasch analysis was used to assess the validity. Differences between groups were evaluated using analysis of variance (ANOVA) and paired *t*-test. The correlation between items was measured by Pearson’s correlation coefficient and the internal consistency was analysed by the Cronbach’s alpha coefficient. The reproducibility was assessed by the Guttman split-half correlation and the intraclass correlation coefficient (ICC) to evaluate reliability.

## Results

A total of 150 people, ranging in age from 18 to 88 years old (mean ±SD, 62.8 ±13.1 years), participated in the study and 56.7% (n = 85) were women (Table 1). Among these, 30 participants were enrolled as normal control (VA levels of 20/20–20/25 in the better eye). The 120 patients with a series of common ophthalmic diseases were recruited and subdivided into 4 subject groups. According to Brown vision level classification [11], 42.5% had good reading vision, 25.8% had legal driving vision; while moderate visual loss has revealed in 25.0% and legal blindness in 6.7%. Group 1 consisted of 30 known cataract patients and 40% of them had VA in the better eye of ≤ 20/50. Group 2 consisted of 30 patients who were classified as wet AMD and 30% of them had VA in the better eye of ≤ 20/50. Group 3 consisted of 30 known diabetic retinopathy patients who have been diagnosed with eitherproliferative (PDR), non-proliferative diabetic retinopathy (NPDR), or diabetic macular edema (DME), and 33.4% had VA in the better eye of ≤ 20/50. Group 4 consisted of 30 glaucoma patients and 23.4% of them had VA of ≤ 20/50.

**Table 1:** The demographic characteristics of participants, mean subscale and composite score of IVI in all subjects.

Participants		Normal	Cataracts	AMD	Diabetic Retinopathy	Glaucoma
**No.**	** **	30	30	30	30	30
**Age range (yr.)**		41–70	32–88	47–85	32–80	18–85
	(mean ± S.D.)	52.8 ± 6.7	69.4 ± 11.8	69.7 ± 9.5	62.5 ± 9.3	59.8 ± 17.6
**Gender (male; No.,%)**		8 (26.7)	11 (36.7)	21 (70.0)	11 (36.7)	14 (46.7)
**Visual acuity, logMAR (range)**		0.0–0.1	0.0–0.7	0.0–1.0	0.0–1.3	0.0–1.0
	(mean ± S.D.)	0.02 ± 0.04	0.29 ± 0.16	0.31 ± 0.29	0.27 ± 0.28	0.24 ± 0.25
**Vision impairment (No., %)**						
	Good reading vision (20/20–20/25)	30 (100)	8 (26.7)	15 (50.0)	14 (46.7)	14 (46.7)
	Legal driving vision (20/30–20/40)	0	10 (33.3)	6 (20.0)	6 (20.0)	9 (30.0)
	Moderate visual loss (20/50–20/100)	0	12 (40.0)	5 (16.7)	8 (26.7)	5 (16.7)
	Legal blindness (≤ 20/200)	0	0	4 (13.3)	2 (6.7)	2 (6.7)
**Summary IVI scales**						
	Reading and accessing information	3.83 ± 1.13	13.30 ± 1.76^†^	14.07 ± 1.67^†^	11.70 ± 1.33^†^	13.00 ± 1.97^†^
	Mobility and independence	3.20 ± 0.73	13.97 ± 1.90^†^	12.67 ± 1.51^†^	14.07 ± 1.70^†^	11.03 ± 1.58^†^
	Emotional well-being	0.83 ± 0.37	5.47 ± 1.17^†^	7.00 ± 1.06^†^	5.93 ± 1.30^†^	4.37 ± 0.96^†^
	Composite score	7.87 ± 2.01	32.73 ± 4.32^†^	33.73 ± 3.53^†^	31.70 ± 3.31^†^	28.40 ± 3.74^†^
**Rasch-transformed IVI scores (in logits)**						
	Reading and accessing information	-1.49 ± 1.84	-0.21 ± 2.41**	0.18 ± 1.51***	-0.07 ± 1.83***	-0.21 ± 2.17**
	Mobility and independence	-2.20 ± 1.76	-0.58 ± 2.25***	-0.20 ± 1.41***	-0.21 ± 1.81***	-1.11 ± 1.92**
	Emotional well-being	-2.86 ± 1.97	-1.67 ± 2.52*	-0.96 ± 2.16***	-1.47 ± 3.04**	-2.17 ± 2.27
	Composite score	-1.01 ± 1.44	-0.20 ± 1.58***	0.54 ± 1.14***	0.36 ± 1.45***	-0.09 ± 1.42**

AMD: Age-related macular degeneration. Visual acuity, log(MAR) in the better eye. Data are expressed at the mean ± SD.

^†^ Significant differences between this group and normal at baseline (P-value < 0.001).

***, **, and * denote statistical significance level of the difference from the reference (Normal) group, at 1%, 5%, and 10% respectively.

The primary objective of this study was the evaluation of the reliability and construct validity of the IVI in native Thai populations. All of the 28 items were considered relevant by 90% or more of the AMD, diabetic retinopathy and glaucoma participants, while the reading ordinary size print and getting information items were revealed 86.7% and 76.7%, respectively in cataracts (S3 Table). The highest response for “don't do this for other reasons than eyesight” was identified in the questions regarding the reading and accessing information items; reading ordinary size print (5.6%) and getting information that you need (7.2%). More than 16.7% of responses for all items were greater than 0 (not at all) and all had responses across the full 0-to-3 range, suggesting that the data was not strongly skewed. The composite and all subscales scores were significantly higher in all vision impairment groups compared with normal group (*P*-value < 0.001, Table 1). The Thai-version IVI showed high criterion validity, with vision impairment participants associated with worse scores on all three IVI subscales (Table 1). The reading and accessing information subscale displayed reasonable fit to the Rasch model with marginal misfit, suggesting unidimensionality within the scale. Two items (items 2 and 4) in the mobility and independence subscale demonstrated misfit (Infit MnSq 1.55 and 1.32, respectively). Upon removal of item 2 (“taking part in recreational activities such as walking, jogging, patong or aerobics”), multidimensionality was resolved. In the reading and accessing information subscale, item 21 (“have you felt embarrassed because of your eyesight”) displayed misfit (Infit MnSq 1.53) and deletion of this item resulting in the scale achieved good fit to the Rasch model. In this study, the differential item functioning (DIF) was tested across subgroups of age (above/below median) and gender (male/female). DIF revealed minor relevance for most items which shows that the items function equivalently for participants independent of their age and gender. The distribution of item difficulties closely matched the distribution of person abilities. All comparisons of vision impairment groups and normal control were significant, except for glaucoma group for the emotional well-being subscale. The evaluation of the reliability of the Thai-version IVI questionnaire is presented in Table 2. Sixty participants including 30 cataracts and 30 normal participated in the reliability studies, with two IVI questionnaires administered 3 to 4 weeks apart (test–retest). For most items in the IVI questionnaire, the difference between item responses was within 1 step in 92% or more cases for both groups. The mean absolute difference scores was less than 1 for each of the subscales and composite IVI score, strong Pearson correlation was detected between test and retest (*r >* 0.72). The Guttman split-half correlation between items in each group was 0.83 for the cataracts and 0.99 for the normal group (data not shown). The intraclass correlation coefficient were 0.956 and 0.969 in cataracts and normal groups, respectively (data not shown). The Cronbach’s alpha coefficient were 0.787, 0.849, and 0.839 for the “reading and accessing information”, “mobility and independence” and “emotional well-being” subscales, respectively, suggesting high internal consistency.

**Table 2:** Person measures for IVI overall and three subscale scores for the normal control and cataract patients.

Person Measures	Control Group					Cataracts Group			
	Absolutedifference	% ≤1 stepdifference	*r*	Cronbach α	Absolutedifference	% ≤1 stepdifference	*r*	Cronbach α
Reading and accessing information	0.11±0.34	99.3	0.91	0.98	0.29±0.63	94.2	0.73	0.79
Mobility and independence	0.06±0.24	100	0.91	0.97	0.28±0.66	92.0	0.86	0.85
Emotional well-being	0.04±0.20	100	0.94	0.97	0.27±0.66	93.3	0.72	0.84
Overall score	0.11±0.34	99.3	0.98	0.99	0.28±0.65	93.1	0.78	0.83

r; Pearson correlation. Data are expressed at the mean ± SD.

The mean composite IVI scores ranged from 28.4 ± 3.7 for the glaucoma group to 33.7 ± 3.5 for the AMD group, while control normal subjects presented mean overall IVI score of 7.9 ± 2.0 (Table 1). The analysis on each subscale score among the four vision defects subgroups revealed no significant differences in the scores of either “mobility and independence”, “emotional well-being” or “reading and accessing information”. The mean scores of each IVI items for the different vision defects groups are all listed in Table 3. Higher values indicate lower visual ability and suggest that the subject is more disabled. All items values ≥ 2 were rated as causing concern or difficulty for vision impairment participants. One item in the reading and accessing information subscale (getting information that you need) was reported to trouble cataract participants (mean ± SD, 2.43 ± 0.58). The item of reading labels or instructions on medicines was rated difficult in AMD and glaucoma patients (mean ± SD = 2.23 ± 0.40 and 2.00 ± 0.36, respectively). In addition, the item of shopping (finding what you want and paying for it) was also reported in glaucoma patients (mean ± SD, 2.00 ± 0.47). None of the items were rated difficult according to the vision related difficulty by diabetic retinopathy participants, the mean score for 28 items ranged from 0.37 to 1.77 in this group.

**Table 3:** Items and subscale scores.

Items	Cataracts		AMD		Diabetic Retinopathy		Glaucoma	
	Score>0, (%)	Mean±SD	Score>0, (%)	Mean±SD	Score>0, (%)	Mean±SD	Score>0, (%)	Mean±SD
1. (R) Watching and enjoying TV	82.8	1.57±0.27	83.3	1.47±0.17	72.4	1.47±0.28	62.1	1.47±0.31
2. (M) Recreational activities	85.2	1.77±0.43	69.0	1.17±0.28	64.3	1.40±0.36	53.6	1.57±0.39
3. (R) Shopping	53.3	1.00±0.20	76.7	1.40±0.17	70.0	1.17±0.17	57.7	**2.00±0.47**
4. (M) Visiting friends or family	64.3	1.47±0.37	73.3	1.27±0.19	71.4	1.77±0.36	46.4	1.33±0.38
5. (R) Recognizing or meeting people	73.3	1.23±0.18	70.0	1.30±0.19	80.0	1.57±0.19	53.3	1.17±0.23
6. (R) Looking after appearance	50.0	0.93±0.20	73.3	1.23±0.18	70.0	1.30±0.19	50.0	0.97±0.21
7. (R) Opening packaging	66.7	1.27±0.20	75	1.70±0.35	63.3	1.17±0.20	50.0	1.10±0.23
8. (R) Reading medical labels	72.4	1.63±0.30	81.5	**2.23±0.40**	70.0	1.37±0.21	78.6	**2.00±0.36**
9. (R) Operating housework	62.1	1.33±0.30	73.3	1.27±0.17	70.0	1.33±0.19	51.7	1.27±0.31
10. (M) Interfered with getting outdoors	66.7	1.33±0.21	82.8	1.40±0.27	79.3	1.77±0.29	58.6	1.40±0.31
11. (M) Avoid falling or tripping	63.3	1.40±0.23	73.3	1.23±0.19	76.7	1.57±0.21	56.7	1.03±0.19
12. (M) Travelling or using transport	60.7	1.53±0.37	69.0	1.30±0.29	69.0	1.53±0.30	42.9	1.23±0.38
13. (M) Going down steps, stairs, or curbs	67.9	1.90±0.37	80.0	1.17±0.15	75.9	1.73±0.29	55.2	1.17±0.30
14. (R) Reading ordinary size print	61.5	1.90±0.47	81.5	1.73±0.40	69.0	1.13±0.27	78.6	1.60±0.34
15. (R) Getting information	52.2	**2.43±0.58**	85.2	1.73±0.40	72.4	1.20±0.27	71.4	1.43±0.35
16. (M) Safety at home	53.3	0.83±0.16	70.0	1.10±0.17	60.0	0.97±0.18	43.3	0.67±0.17
17. (M) Spilling or breaking things	46.7	0.80±0.19	60.0	0.83±0.14	43.3	0.60±0.15	50.0	0.63±0.13
18. (M) Safety outside the home	63.3	1.20±0.21	76.7	1.27±0.18	73.3	1.07±0.16	50.0	0.77±0.16
19. (M) Stopped doing the things	46.7	0.93±0.21	63.3	1.07±0.20	53.3	0.77±0.16	43.3	0.60±0.14
20. (M) Needed help from other people	56.7	0.80±0.16	63.3	0.87±0.16	56.7	0.90±0.19	40.0	0.63±0.17
21. (E) Felt embarrassed	26.7	0.43±0.16	30.0	0.50±0.16	23.3	0.37±0.14	16.7	0.23±0.10
22. (E) Felt frustrated or annoyed	40.0	0.67±0.18	53.3	0.87±0.18	40.0	0.73±0.19	46.7	0.63±0.14
23. (E) Felt lonely or isolated	26.7	0.43±0.16	30.0	0.47±0.15	26.7	0.50±0.18	23.3	0.37±0.14
24. (E) Felt sad or low	26.7	0.40±0.14	40.0	0.60±0.16	36.7	0.63±0.18	30.0	0.37±0.11
25. (E) Worried about eyesight worsen	53.3	1.00±0.20	76.7	1.33±0.18	53.3	1.07±0.22	50.0	0.97±0.20
26. (E) Worried about coping with everyday life	53.3	0.87±0.19	66.7	1.17±0.19	46.7	0.90±0.21	46.7	0.63±0.15
27. (E) Felt like a nuisance or a burden	50.0	0.80±0.18	53.3	1.00±0.20	46.7	0.83±0.19	40.0	0.63±0.17
28. (E) Interfered with life in general	53.3	0.87±0.18	70.0	1.07±0.17	53.3	0.90±0.18	33.3	0.53±0.16

R, reading and accessing information; M, mobility and independence; E, emotional well-being.

## Discussion

Many instruments attempted to qualify VRQoL by assessing subjective difficulties of the daily activities. However, the use of a cheap, easy and reliable instrument is arguably more important in developing countries and among individuals with low socioeconomic status. Our study showed that the Thai-version of the IVI questionnaire was of adequate reliability and validity to be used for the evaluation VRQoL of many ophthalmic disorders. Obviously, the performance of QoL instruments is dependent on the proper adaptation of the items to the cultural characteristics of the population studied. In Thai-version IVI, the proper minor modifications were applied to adapt the instrument to the Thai setting. Minor adaptations of some items were also considered necessary during the translation and validation of the IVI in other populations [8,12]. Depending on the subjects' responses, each item of the IVI was given a score from 0 to 3, where higher scores indicated poorer VRQoL. Relatively low relevant rates (90% relevant of 28 items) were encountered in this population. The criterion validity of the Thai-version IVI was demonstrated by its ability to significantly discriminate between vision impairment patients and normal participants. In addition, the subscales of the Thai-version IVI questionnaire presented variable but high internal consistencies (Cronbach's alpha = 0.787–0.849) indicating high reliability of the instrument in the test-retest population studied. According to traditional validation methods, hence, the results of this clinical-based study shows that the Thai-version IVI questionnaire is a valid and reliable instrument for the assessment of VSQoL in the native populations.

Visual impairment is a global concern on a social, economic and personal level. A series of VSQoL studies conducted in homogenous populations have assessed the impact of impaired vision diseases on VSQoL [13,14]. However, most of the studies were performed in Western countries or in urbanized regions. There are often marked variations between different geographical areas, socio-economics groups, and age and gender in terms of prevalence of impaired vision and coverage by eye care services. In our study, we tried to include all of the major visual impairment diseases to investigate their impact on the Thai patients VSQoL. Moreover, we carried out our study in a remote rural region with a relatively low degree of technical development. Cataract and glaucoma are among the main causes of vision defects and blindness in Thailand [15,16]. Cataract is one of the age-related diseases that is often responsible for vision impairment in an aging population [17]. In addition, AMD and diabetic retinopathy are also common vision impairment causes presented in Thailand [18]. AMD is currently the major cause of severe vision loss in people aged 65 years or over in the developed world that estimated to be about 10% between 65 and 75 years and increases threefold in those over 75 years [19,20]. AMD-related vision impairment has been associated with depression, poor mental health and reduced QoL [21,22]. Hence, the high population growth and increasing number of elderly people in Thailand contribute to the upward trend of these diseases. Although, our study is limited by a small sample size and included several patients with no significant visual defect when based on better eye visual acuity. The results showed that all participants have no concern in the mobility and independence and the emotional well-being subscales while patients with cataract, AMD and glaucoma are reported to be concern in the reading and accessing information subscale. Most of cataracts, AMD and glaucoma patients who are concerned in the reading and accessing information subscale are chronic and have severely impaired vision. On the other hand, diabetic retinopathy patients were not concern on any vision related difficulty because the majority of the participated patients were diagnosed as having mild to moderate vision impairment. Although the correlations between visual acuity of the subjects and IVI questionnaire have been detected during the validation in other populations [7], additional studies are needed to address these questions in Thai population. In addition to socio-economics compatibility, our results suggested that the Thai-version IVI questionnaire has sufficient impact to evaluate the VRQoL in Thai visual impairment patients. This study indicated that the symptoms of the common chronic eye diseases are associated with an adverse impact on VRQoL. Furthermore, the findings in this study add further insight into the consideration of vision defects as a significant public health problem that deserves further study to improve the QoL of the general population. However, one limitation of our study was that only of individuals presenting in rural hospital were enrolled, so it is questionable whether this questionnaire could be used for population screening purposes or relevant to urban population.

## Supporting Information

S1 Table The specific items of the IVI questionnaire. (PDF)

S2 Table Thai version IVI questionnaire. (PDF)

S3 Table Questionnaire items and scoring characteristics in Thai patients with visual defects. (PDF)
