# Supplementary material for: The Impact of Vision Impairment (IVI) Questionnaire; Validation of the Thai-Version and the Implementation on Vision-Related Quality of Life in Thai Rural Community
Source: PLoS One. 2016 May 18;11(5):e0155509. doi: 10.1371/journal.pone.0155509 (PMC4871442; doi:10.1371/journal.pone.0155509)
Supplement: S1 Table — (PDF) [file pone.0155509.s001.pdf]

Impact of Vision Impairment Profile (IVI)

Thai version

(Translated and Modified from the Centre for Eye Research Australia (CERA) 2007)

INSTRUCTIONS

Please read each question carefully and circle the answer that BEST applies to you.

Put one circle on each row.

If you use GLASSES, CONTACT LENSES OR MAGNIFIERS for some activities please answer according to how you can see when using them.

Here are two examples:

In the PAST MONTH, how often has YOUR EYESIGHT MADE YOU CONCERNED OR WORRIED about the following:

	Not at all	A little	A fair amount	A lot	Don't do this for other reasons
Crossing the street?	0	1	2	3	8
Preparing a meal for yourself?	0	1	2	3	8

PLEASE START HERE AND REMEMBER:

Put one circle on each row. Please do not leave any rows blank.

Please answer about YOUR eyesight with GLASSES, CONTACT LENSES, or MAGNIFIERS, if you use them.

In the PAST MONTH, how much has YOUR EYESIGHT INTERFERED with the following activities:

	Not at all	A little	A fair amount	A lot	Don't do this for other reasons	Domains
1. Your ability to see and enjoy T.V.?	0	1	2	3	8	Reading and accessing information
2. Taking part in recreational activities such as walking, jogging, patong or aerobics?	0	1	2	3	8	Mobility and independence
3. Shopping? (finding what you want and paying for it)	0	1	2	3	8	Reading and accessing information
4. Visiting friends or family?	0	1	2	3	8	Mobility and independence
5. Recognising or meeting people?	0	1	2	3	8	Reading and accessing information
6. Generally looking after your appearance? (face, hair, clothing, etc.)	0	1	2	3	8	Reading and accessing information
7. Opening packaging? (for example, around food, medicines)	0	1	2	3	8	Reading and accessing information

Please answer about YOUR eyesight with GLASSES, CONTACT LENSES, or MAGNIFIERS, if you use them.

In the PAST MONTH, how much has YOUR EYESIGHT INTERFERED with the following activities:

	Not at all	A little	A fair amount	A lot	Don't do this for other reasons	Domains
8. Reading labels or instructions on medicines?	0	1	2	3	8	Reading and accessing information
9. Operating household appliances and the telephone?	0	1	2	3	8	Reading and accessing information
10. How much has your eyesight interfered with getting about outdoors? (on the pavement or crossing the street)	0	1	2	3	8	Mobility and independence
11. In the past month, how often has your eyesight made you go carefully to avoid falling or tripping?	0	1	2	3	8	Mobility and independence
12. In general, how much has your eyesight interfered with travelling or using transport? (bus & train)	0	1	2	3	8	Mobility and independence
13. Going down steps, stairs, or curbs?	0	1	2	3	8	Mobility and independence

Please answer about YOUR eyesight with GLASSES, CONTACT LENSES, or MAGNIFIERS, if you use them.

In the PAST MONTH, how much has YOUR EYESIGHT INTERFERED with the following activities:

	Not at all	A fair amount	A lot	Don't do this for other reasons	Domains
14. Reading ordinary size print? (for example newspapers)	0	1	2	8	Reading and accessing information
15. Getting information that you need?	0	1	2	8	Reading and accessing information

Please answer about YOUR eyesight with GLASSES, CONTACT LENSES or MAGNIFIERS, if you use them.

In the PAST MONTH, how often has YOUR EYESIGHT MADE YOU CONCERNED OR WORRIED about the following:

	Not at all	A little of the time	A fair amount of the time	A lot of the time	Domains
16. Your general safety at home?	0	1	2	3	Mobility and independence
17. Spilling or breaking things?	0	1	2	3	Mobility and independence
18. Your general safety when out of your home?	0	1	2	3	Mobility and independence
19. In the past month, how often has your eyesight stopped you doing the things you want to do?	0	1	2	3	Mobility and independence
20. In the past month, how often have you needed help from other people because of your eyesight?	0	1	2	3	Mobility and independence

Please answer about YOUR eyesight with GLASSES, CONTACT LENSES or MAGNIFIERS, if you use them.

Think about how YOUR eyesight has made you FEEL in the PAST MONTH.

	Not at all	A little of the time	A fair amount of the time	A lot of the time	Domains
21. Have you felt embarrassed because of your eyesight?	0	1	2	3	Emotional well-being
22. Have you felt frustrated or annoyed because of your eyesight?	0	1	2	3	Emotional well-being
23. Have you felt lonely or isolated because of your eyesight?	0	1	2	3	Emotional well-being
24. Have you felt sad or low because of your eyesight?	0	1	2	3	Emotional well-being
25. In the past month, how often have you worried about your eyesight getting worse?	0	1	2	3	Emotional well-being
26. In the past month how often has your eyesight made you concerned or worried about coping with everyday life?	0	1	2	3	Emotional well-being
27. Have you felt like a nuisance or a burden because of your eyesight?	0	1	2	3	Emotional well-being
28. In the past month, how much has your eyesight interfered with your life in general?	0	1	2	3	Emotional well-being

Please check that you have answered all the questions and Thank you!
