# Supplementary material for: The Impact of Vision Impairment (IVI) Questionnaire; Validation of the Thai-Version and the Implementation on Vision-Related Quality of Life in Thai Rural Community
Source: PLoS One. 2016 May 18;11(5):e0155509. doi: 10.1371/journal.pone.0155509 (PMC4871442; doi:10.1371/journal.pone.0155509)
Supplement: S2 Table — (PDF) [file pone.0155509.s002.pdf]

แบบทดสอบประเมินผลกระทบของความผิดปกติในการมองเห็น

(แปลจากแบบทดสอบของศูนย์วิจัยทางจักษุวิทยา ประเทศออสเตรเลีย 2550)

<u>คำแนะนำ</u>

กรุณาอ่านคำถามอย่างรอบคอบและทำเครื่องหมายวงกลม O รอบคำตอบที่ตรงกับท่านมากที่สุด

วงกลมเพียงครั้งเดียวในแต่ละแถว

หากท่านสวมแว่นตา คอนแทคเลนส์ หรืออุปกรณ์ช่วยขยาย ในการทำกิจกรรมบางอย่าง กรุณาเลือกคำตอบตามที่ท่านสามารถมองเห็นขณะใส่แว่นตาหรือคอนแทคเลนส์

ต่อไปนี้คือตัวอย่าง 2 ข้อ

<u>ในเดือนที่ผ่านมา บ่อยครั้งเพียงใดที่การมองเห็นของท่านทำให้ท่านมีความรู้สึกวิตกกังวลเกี่ยวกับสิ่งต่อไปนี้</u>

	ไม่เลย	เล็กน้อย	ปานกลาง	มาก	ไม่ได้ทำกิจกรรมนี้ด้วยเหตุผลอื่น
ข้ามถนน	**0**	**1**	**2**	**(3)**	**8**
จัดเตรียมอาหารด้วยตนเอง	**0**	**1**	**(2)**	**3**	**8**

เริ่มทำตรงนี้ และโปรดจำ

ทำเครื่องหมายวงกลมเพียงหนึ่งวงในแต่ละแถวและโปรดอย่าปล่อยให้มีแถวว่าง

เลือกคำตอบตามที่ท่านมองเห็นขณะใส่แว่นตา คอนแทคเลนส์ หรืออุปกรณ์ช่วยขยาย (ถ้าใช้)

<u>ในเดือนที่ผ่านมา บ่อยครั้งเพียงใดที่การมองเห็นของท่านรบกวนการทำกิจกรรมต่อไปนี้</u>

	ไม่เลย	เล็กน้อย	ปานกลาง	มาก	ไม่ได้ทำกิจกรรมนี้ด้วยเหตุผลอื่น (ที่ไม่เกี่ยวกับการมองเห็น)	ขอบเขตการประเมิน
**1.** การรับชมและเพลิดเพลินกับรายการโทรทัศน์	**0**	**1**	**2**	**3**	**8**	การอ่านและการเข้าถึงข้อมูล
**2.** การเข้าร่วมกิจกรรมสันทนาการ เช่น การเดินเล่น วิ่งจ๊อกกิ้ง เปตอง หรือเต้นแอโรบิค	**0**	**1**	**2**	**3**	**8**	การเคลื่อนไหวและไม่ต้องพึ่งพาผู้อื่น
**3.** การจับจ่ายซื้อของ (เลือกหาสิ่งที่ต้องการและจ่ายเงินซื้อ)	**0**	**1**	**2**	**3**	**8**	การอ่านและการเข้าถึงข้อมูล
**4.** เยี่ยมเยียนเพื่อนฝูงและญาติ	**0**	**1**	**2**	**3**	**8**	การเคลื่อนไหวและไม่ต้องพึ่งพาผู้อื่น
**5.** การจดจำทักทายและพบปะผู้คน	**0**	**1**	**2**	**3**	**8**	การอ่านและการเข้าถึงข้อมูล
**6.** การดูแลภาพลักษณ์ของตนเองโดยทั่วไป (แต่งหน้า หวีผม จัดแต่งเสื้อผ้า และอื่น ๆ)	**0**	**1**	**2**	**3**	**8**	การอ่านและการเข้าถึงข้อมูล
**7.** เปิดบรรจุภัณฑ์ (เช่น อาหาร ยา)	**0**	**1**	**2**	**3**	**8**	การอ่านและการเข้าถึงข้อมูล

เลือกคำตอบตามที่ท่านมองเห็นขณะใส่แว่นตา คอนแทคเลนส์ หรืออุปกรณ์ช่วยขยาย (ถ้าใช้)

ในเดือนที่ผ่านมา บ่อยครั้งเพียงใดที่การมองเห็นของท่านรบกวนการทำกิจกรรมต่อไปนี้

	ไม่เลย	เล็กน้อย	ปานกลาง	มาก	ไม่ได้ทำกิจกรรมนี้ด้วยเหตุผลอื่น (ที่ไม่เกี่ยวกับการมองเห็น)	ขอบเขตการประเมิน
**8.** อ่านฉลากและคำแนะนำการใช้ยา	**0**	**1**	**2**	**3**	**8**	การอ่านและการเข้าถึงข้อมูล
**9.** ใช้งานอุปกรณ์เครื่องใช้ในบ้านและโทรศัพท์	**0**	**1**	**2**	**3**	**8**	การอ่านและการเข้าถึงข้อมูล
**10.** การมองเห็นของท่านรบกวนการออกไปใช้ชีวิตนอกบ้านมากน้อยเพียงใด (การเดินทางเท้า การข้ามถนน)	**0**	**1**	**2**	**3**	**8**	การเคลื่อนไหวและไม่ต้องพึ่งพาผู้อื่น
**11.** ในเดือนที่ผ่านมา สภาพการมองเห็นของท่านทำให้ท่านต้องระมัดระวังการสะดุดหกล้มมากน้อยเพียงใด	**0**	**1**	**2**	**3**	**8**	การเคลื่อนไหวและไม่ต้องพึ่งพาผู้อื่น
**12.** โดยทั่วไป การมองเห็นของท่านรบกวนการเดินทางท่องเที่ยวหรือโดยสารพาหนะ (เช่น รถเมล์ รถไฟ) มากน้อยเพียงใด	**0**	**1**	**2**	**3**	**8**	การเคลื่อนไหวและไม่ต้องพึ่งพาผู้อื่น
**13.** การก้าวลงขั้นบันได ทางต่างระดับ หรือขอบทางเดิน	**0**	**1**	**2**	**3**	**8**	การเคลื่อนไหวและไม่ต้องพึ่งพาผู้อื่น

เลือกคำตอบตามที่ท่านมองเห็นขณะใส่แว่นตา คอนแทคเลนส์ หรืออุปกรณ์ช่วยขยาย (ถ้าใช้)

ในเดือนที่ผ่านมา บ่อยครั้งเพียงใดที่การมองเห็นของท่านรบกวนการทำกิจกรรมต่อไปนี้

	ไม่เลย	พอสมควร	มาก	ไม่ได้ทำกิจกรรมนี้ด้วยเหตุผลอื่น (ที่ไม่เกี่ยวกับการมองเห็น)	ขอบเขตการประเมิน
**14.** อ่านตัวพิมพ์ขนาดปกติ (เช่นหนังสือพิมพ์)	0	1	2	8	การอ่านและการเข้าถึงข้อมูล
**15.** ค้นหาข้อมูลที่ต้องการ	0	1	2	8	การอ่านและการเข้าถึงข้อมูล

เลือกคำตอบตามที่ท่านมองเห็นขณะใส่แว่นตา คอนแทคเลนส์ หรืออุปกรณ์ช่วยขยาย (ถ้าใช้)

ในเดือนที่ผ่านมา บ่อยครั้งเพียงใดที่การมองเห็นของท่านทำให้ท่านมีความรู้สึกวิตกกังวลเกี่ยวกับสิ่งต่อไปนี้

	ไม่เลย	น้อยครั้ง	พอสมควร	บ่อยครั้งมาก	ขอบเขตการประเมิน
**16.** ความปลอดภัยโดยทั่วไปในการใช้ชีวิตภายในบ้าน	0	1	2	3	การเคลื่อนไหวและไม่ต้องพึ่งพาผู้อื่น
**17.** การทำของหกหรือทำสิ่งของตกแตก	0	1	2	3	การเคลื่อนไหวและไม่ต้องพึ่งพาผู้อื่น
**18.** ความปลอดภัยโดยทั่วไปเวลาใช้ชีวิตนอกบ้าน	0	1	2	3	การเคลื่อนไหวและไม่ต้องพึ่งพาผู้อื่น
**19.** ในเดือนที่ผ่านมา บ่อยครั้งเพียงใดที่การมองเห็นของท่านเป็นอุปสรรคขัดขวางทำให้ท่านต้องล้มเลิกการทำสิ่งที่ท่านต้องการทำ	0	1	2	3	การเคลื่อนไหวและไม่ต้องพึ่งพาผู้อื่น
**20.** ในเดือนที่ผ่านมา บ่อยครั้งเพียงใดที่ท่านต้องการความช่วยเหลือจากผู้อื่นเนื่องจากปัญหาการมองเห็นของท่าน	0	1	2	3	การเคลื่อนไหวและไม่ต้องพึ่งพาผู้อื่น

เลือกคำตอบตามที่ท่านมองเห็นขณะใส่แว่นตา คอนแทคเลนส์ หรืออุปกรณ์ช่วยขยาย (ถ้าใช้)

<u>ให้นึกถึงความรู้สึกที่ท่านมีต่อการมองเห็นของตนเองในเดือนที่ผ่านมา</u>

	ไม่เลย	น้อยครั้ง	พอสมควร	บ่อยครั้งมาก	ขอบเขตการประเมิน
**21.** ท่านรู้สึกอับอายกับความสามารถในการมองเห็นของตนเองหรือไม่	**0**	**1**	**2**	**3**	สภาวะอารมณ์
**22.** ท่านรู้สึกท้อแท้หรือรำคาญเนื่องจากความสามารถในการมองเห็นของตนเองหรือไม่	**0**	**1**	**2**	**3**	สภาวะอารมณ์
**23.** ท่านรู้สึกเปล่าเปลี่ยวหรือโดดเดี่ยวอันมีสาเหตุเนื่องจากความสามารถในการมองเห็นของตนเองหรือไม่	**0**	**1**	**2**	**3**	สภาวะอารมณ์
**24.** ท่านรู้สึกเศร้าเสียใจหรือรู้สึกด้อยค่าอันมีสาเหตุเนื่องจากความสามารถในการมองเห็นของตนเองหรือไม่	**0**	**1**	**2**	**3**	สภาวะอารมณ์
**25.** ในเดือนที่ผ่านมา ท่านรู้สึกวิตกกังวลว่าความสามารถในการมองเห็นของตนเองจะแย่ลงบ่อยครั้งเพียงใด	**0**	**1**	**2**	**3**	สภาวะอารมณ์
**26.** ในเดือนที่ผ่านมา ความสามารถในการมองเห็นของท่านทำให้ท่านรู้สึกวิตกกังวลเกี่ยวกับการจัดการเรื่องราวต่าง ๆในชีวิตประจำวันบ่อยครั้งเพียงใด	**0**	**1**	**2**	**3**	สภาวะอารมณ์
**27.** ท่านรู้สึกว่าตนเองเป็นภาระต้องรบกวนผู้อื่นเนื่องจากความสามารถในการมองเห็นของตนเองหรือไม่	**0**	**1**	**2**	**3**	สภาวะอารมณ์
**28.** ในเดือนที่ผ่านมา ความสามารถในการมองเห็นของท่านรบกวนการดำเนินชีวิตโดยรวมมากน้อยเพียงใด	**0**	**1**	**2**	**3**	สภาวะอารมณ์

<u>กรุณาตรวจสอบว่าท่านได้ตอบครบทุกคำถาม ขอขอบพระคุณ</u>
