# Supplementary material for: The Impact of Vision Impairment (IVI) Questionnaire; Validation of the Thai-Version and the Implementation on Vision-Related Quality of Life in Thai Rural Community
Source: PLoS One. 2016 May 18;11(5):e0155509. doi: 10.1371/journal.pone.0155509 (PMC4871442; doi:10.1371/journal.pone.0155509)
Supplement: S3 Table — (PDF) [file pone.0155509.s003.pdf]

Additional file 3. Questionnaire items and scoring characteristics in Thai patients with visual defects.

Items	Cataracts		AMD		Diabetic Retinopathy		Glaucoma	
	Range, min/max	Relevant, (%)	Range, min/max	Relevant, (%)	Range, min/max	Relevant, (%)	Range, min/max	Relevant, (%)
1. (R) Watching and enjoying TV	0/3	96.7	0/3	100	0/3	96.7	0/3	96.7
2. (M) Recreational activities	0/3	90.0	0/3	96.7	**0/2**	93.3	0/3	93.3
3. (R) Shopping	0/3	100	0/3	100	0/3	100	0/3	86.7
4. (M) Visiting friends or family	0/3	93.3	0/3	100	0/3	93.3	0/3	93.3
5. (R) Recognizing or meeting people	0/3	100.0	0/3	100	0/3	100	0/3	100
6. (R) Looking after appearance	0/3	100	0/3	100	0/3	100	0/3	100
7. (R) Opening packaging	0/3	100	0/3	93.3	0/3	100	0/3	100
8. (R) Reading medical labels	0/3	96.7	0/3	90.0	0/3	100	0/3	93.3
9. (R) Operating housework	0/3	96.7	0/3	100	0/3	100	0/3	96.7
10. (M) Interfered with getting outdoors	0/3	100.0	0/3	96.7	0/3	96.7	0/3	96.7
11. (M) Avoid falling or tripping	0/3	100	0/3	100	0/3	100	0/3	100
12. (M) Travelling or using transport	0/3	93.3	0/3	96.7	0/3	96.7	0/3	93.3
13. (M) Going down steps, stairs, or curbs	0/3	93.3	0/3	100	0/3	96.7	0/3	96.7
14. (R) Reading ordinary size print	0/3	86.7	0/3	90.0	**0/2**	96.7	**0/2**	93.3
15. (R) Getting information	0/3	76.7	0/3	90.0	**0/2**	96.7	**0/2**	93.3
16. (M) Safety at home	**0/2**	100	0/3	100	0/3	100	0/3	100
17. (M) Spilling or breaking things	0/3	100	**0/2**	100	0/3	100	**0/2**	100
18. (M) Safety outside the home	0/3	100	0/3	100	0/3	100	0/3	100
19. (M) Stopped doing the things	0/3	100	0/3	100	0/3	100	**0/2**	100
20. (M) Needed help from other people	0/3	100	0/3	100	0/3	100	0/3	100
21. (E) Felt embarrassed	0/3	100	0/3	100	0/3	100	**0/2**	100
22. (E) Felt frustrated or annoyed	0/3	100	0/3	100	0/3	100	**0/2**	100
23. (E) Felt lonely or isolated	0/3	100	0/3	100	0/3	100	0/3	100
24. (E) Felt sad or low	0/3	100	0/3	100	0/3	100	**0/2**	100
25. (E) Worried about eyesight worsen	0/3	100	0/3	100	0/3	100	0/3	100
26. (E) Worried about coping with everyday life	0/3	100	0/3	100	0/3	100	0/3	100
27. (E) Felt like a nuisance or a burden	0/3	100	0/3	100	0/3	100	0/3	100
28. (E) Interfered with life in general	0/3	100	0/3	100	0/3	100	0/3	100

R, reading and accessing information; M, Mobility and independence; E, emotional well-being.
